# Impact of an IEC (Information, Education and Communication) intervention on key family practices of mothers related to child health in Jamshoro, Sindh

**DOI:** 10.12669/pjms.303.4798

**Published:** 2014

**Authors:** Salma Shaikh, Shazia Memon, Imran Ahmed, Amna, Rabia Manzoor, Saleem Shaikh

**Affiliations:** 1 Dr. Salma Shaikh, Professor of Pediatrics, Liaquat University of Medical & Health Sciences (LUMHS), Jamshoro, Sindh, Pakistan.; 2 Dr. Shazia Memon, Assistant Professor of Pediatrics, Liaquat University of Medical & Health Sciences (LUMHS), Jamshoro, Sindh, Pakistan.; 3 Dr. Imran Ahmed, FCPS, Resident of Pediatrics, Liaquat University of Medical & Health Sciences (LUMHS), Jamshoro, Sindh, Pakistan.; 4 Dr. Amna, Medical officer, Liaquat University of Medical & Health Sciences (LUMHS), Jamshoro, Sindh, Pakistan.; 5 Dr. Rabia Manzoor, Liaquat University of Medical & Health Sciences (LUMHS), Jamshoro, Sindh, Pakistan.; 6 Dr. Saleem Shaikh, Research officer, Liaquat University of Medical & Health Sciences (LUMHS), Jamshoro, Sindh, Pakistan.

**Keywords:** Child health, IEC intervention, Health education, Key family practices, Mother’s practices

## Abstract

***Objective: ***To determine change in practice of mothers having children less than five years of age in five key areas related to child health, growth and development including immunization, feeding during illness, appropriate home treatment for infections and care seeking behavior.

***Methods:*** This was a community based interventional study of Information, Education and Communication (IEC) intervention in the UC Jamshoro, Taluka Kotri, district Jamshoro of 15 months duration from March 2011 to June 2012. Ninety five mothers having children less than five years of age were selected by systematic random sampling for house hold based survey by questionnaire designed by EPP evaluation and health section of UNICEF during baseline and post-intervention phases. Base line data was collected from the interventional area then health education messages were given through written and pictorial material by LHWs for 9 months. To measure the impact helath education messages, data was again collected by same questionnaire are from the same union council during post-intervention phase.

***Results:*** During baseline survey except immunization all other key family practices were poor. After 9 months of intervention of repeated heath education sessions through LHW during their routine visits all practices were improved with statistically significant difference. Regarding the comparison of the results between baseline and post-intervention surveys we found that except immunization which was already better, all those practices which requires mother’s knowledge and practice were improved after our intervention with significant P-values.

***Conclusions:*** Improving the mother’s education level is very important, to empower the first care provider of child in the community. However, in the mean time, health educational messages related to the limited number of key family practices should be disseminated.

## INTRODUCTION

Under five year mortality is about 11 million in developing countries.^1^^,^^2^ Approximately two third are due to five diseases including: pneumonia, diarrhoea, measles, malaria and malnutrition.^3^

In Pakistan the current status of infant mortality is 62 and under-five mortality is 86 deaths per 1,000 live births.^4^ Over half of deaths under five occur during the neonatal period and 26 percent occur during the post-neonatal period.^5^ Although child health in Pakistan has been the subject of much attention for several decades, overall progress in this area has been slow as there was vertical implementation of the programs.

Disease-specific programmes have improved child survival in many developing countries,^4^ but because of their limited focused approach desired reduction in childhood mortality was not achieved. In order to address childhood illnesses in an integrated manner, the World Health Organization (WHO), worked with the United Nation Children's Fund (UNICEF) and other partners to develop a strategy known as Integrated Management of Neonatal and Childhood Illnesses (IMNCI).^1^ The IMNCI has three components: improvement in the case-management practices of health staff through the provision of locally adapted guidelines on IMNCI, improvements in health systems required for effective management of childhood illness, and improvement in household and community practices. After expansion of the first two components, researchers are now focusing on third component: household and community practices (HH/ CP IMNCI).

The objective of house hold and community component is to empower communities to address factors that affect child health,^5^ which could not be achieved unless we explore the existing practices of mothers regarding child health. It has been identified that lack of knowledge and delayed care-seeking contributes in up to 70% of childhood deaths especially in rural settings.^6^

The current challenges in child survival are therefore to improve access to basic knowledge and appropriate quality services for those who need them most. This could be achieved by strengthening IMNCI strategy in the community. IMNCI was introduced in Pakistan in September 1998 when Ministry of Health formally endorsed but implementation phase at district level was started in November 2000 and completed in October 2001. The IMCI community component was launched in Pakistan in March 2002.^7^

Families and communities need to be empowered with knowledge and skills regarding child health and development and communities need to be mobilized and motivated.^8^ This is important because usually the management of sick child starts at home, especially in rural community. Evidence has shown that up to 80% of deaths of children <5 years occurs at home with little or no contact with health providers.^9^

This was community based IEC intervention. The IEC material, Inter Personal Communication, counseling sessions and health education sessions were used to educate the mothers having under 5 years children of union council Jamshoro.

The purpose of our study was to assess the impact of health education through Information, Education and Communication intervention over the knowledge and practices of mothers having at least one child of <5 years of age on following 05 key family practices related to child health:

1. *Immunization:* Take children as scheduled to complete a full course of immunizations (BCG, Pentavalent (DPT, Hib, and Hep-B), OPV and measles) before their first birthday.
2. *Home care for illness:* Continue to feed and offer more fluids, including breast milk, to children when they are sick.
3. *Home treatment for infections:* Give sick children appropriate home treatment for infections.
4. *Care-seeking:* Recognize when sick children need treatment outside the home and seek care from appropriate providers.
5. *Compliance with advice:* Follow the health worker’s advice about treatment, follow-up and referral.

Our goal was to reduce under 5 morbidity and mortality by improving the practices of mothers of the union council Jamshoro, Taluka Kotri district Jamshoro, Sindh. The results of the intervention will help to improve the community based intervention of the IMCI, enable the policy makers to improve the child health policies and planners to prepare and implement effective IEC/BCC programs/projects.

## METHODS

The total population of union council Jamshoro was about 29930 with 53% covered by LHWs. The total number of LHWs were18 supervised by one Lady Health Supervisor. Because of limited resources four out of eighteen LHWs had been selected for IEC intervention. The population covered by the proposed four LHWs was 3801 with <5 years children 603 and the mothers of <5 years children were 378 (LHW’s Khandan Registers). Regarding the sample size only 95 (25% of the 378) was taken keeping in view the convenience and logistic availability. Sample was divided among the four LHWs on the basis of the proportion of the target population in their areas. Before implementation of the intervention the baseline data on 05 key family practices was collected through a Household Baseline Survey questionnaire which was pre-tested in the field. After modification as per results of pre-testing it was translated in Sindhi and used for both pre and post intervention surveys. The sampling technique was systemic random. The first household was selected by random number, then every fifth house hold was surveyed. If a household had no mother of <5 year of age child then next fifth household was visited. The LHS and LHWs were trained in Interpersonal communication and group counseling sessions about the key family practices to be addressed during health education sessions with mothers. The LHWs were closely monitored and supervised by the LHS, Co-investigators and Principle Investigator (PI) in the field during the supervisory visits. After the nine months of IEC intervention the end-line survey was conducted. The baseline and end-line survey findings were analyzed in terms of frequencies, percentages and proportions. PC software packages SPSS version 18 was used for analysis.

## RESULTS

**Fig.1:**
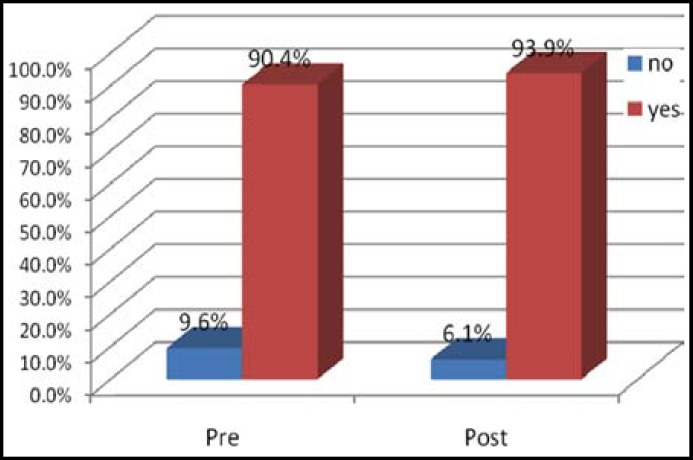
Status of BCG vaccination

During the house hold survey (95 mothers in the baseline and 98 mothers in post intervention) having under 5 year children were interviewed by predesigned questionnaire. To assess the immunization status. Mothers having a child age 12-23 months were asked whether they had a vaccination card for the child. If a card was available, interviewers were required to copy carefully. Otherwise the immunization status was assessed by mother’s recall memory. Practices of keeping the vaccination cards was improved from 40% in the baseline to 68% in the post-intervention survey. Vaccination Status for those who don't have Vaccination Card was also improved after intervention. Regarding BCG immunization coverage was almost more than 90% with no significant difference in pre-and post intervention survey. Status of polio vaccination is shown in Table-I and BCG vaccination in Fig.1.

**Table-I:** Status of polio vaccination N=153

*Whether or not vaccinated*	*Pre-intervention*	*%*	*Post intervention*	*%*	*Total*
No	1	0.96%	1	2.04%	2
Yes	103	99.04%	48	97.96%	151
Total	104		49		153

Fisher’s exact test p= .539

**Fig-2:**
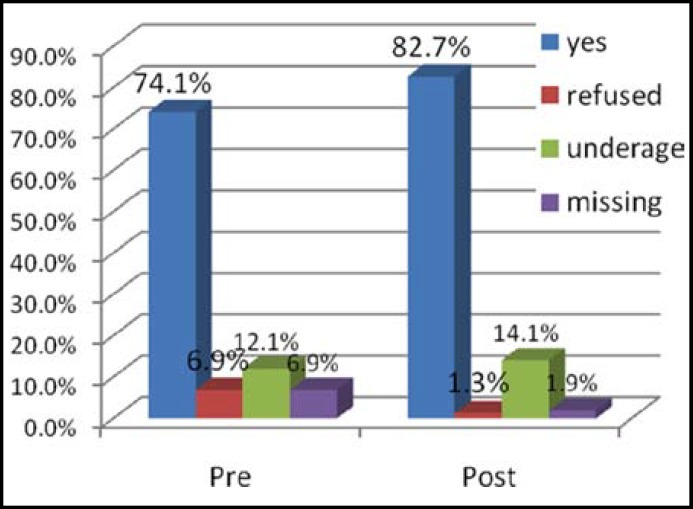
Status of measles 1 vaccination.

**Fig-3:**
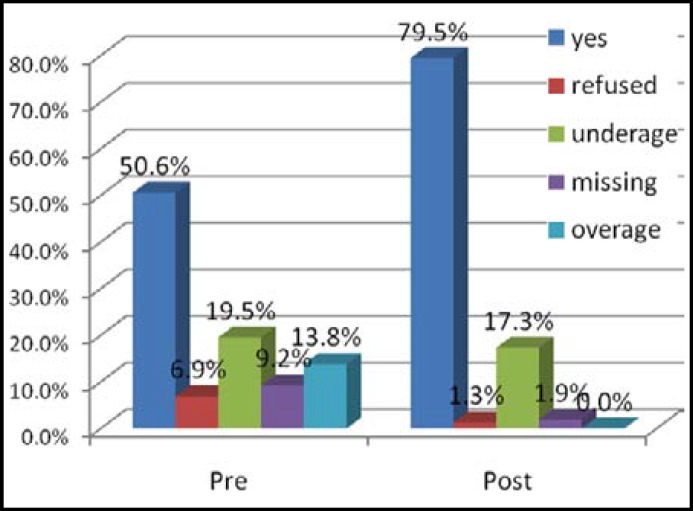
Status of measles 2 vaccination N=330.

The status and coverage of pentavalent vaccine was also improved after intervention from 74% to 95% and coverage upto 3 doses of penta has increased from 57 to 72%, this difference was statistically significant with P-value 0.001. The status of measles immunization was also improved after intervention with statistically significant P-value (<0.05). (Status of Measles immunization is shown in Fig.2 and 3).

Pearson Chi Sqrare p= .009

Pearson Chi square p<.000

Regarding the continued feeding during illness we enquired about the episode of current illness mainly history of pneumonia, diarrhea, fever/ malaria and measles in last 2 weeks. To assess mothers practice of continued feeding we enquired the drinking and eating pattern of children during their sickness. This practice was improved with statistically significant P-value. In Table-II drinking pattern during illness is shown.

**Table-II:** Child drinking pattern during illness N=174

*Drinking pattern*	*Pre intervention*	*%*	*Post intervention*	*%*	*Total*
Much less	61	49.59%	7	13.73%	68
Same	60	48.78%	30	58.82%	90
More than usual	0	0.00%	14	27.45%	14
Don’t know	2	1.63%	0	0.00%	2
Total	123		51		174

Person Chi square p< .000

To assess the mother’s practice of giving appropriate home treatment to their sick children we enquired about the appropriate home treatment practice during last diarrheal episode. The interviewer asked regarding the use of ORS, homemade fluid or and the administration of increased food and fluid during diarrhea. We enquired about breast milk for younger children (<2 years) and other fluids including ORS for older children. Mothers responses is shown in Table-III.

**Table-III:** Practices of mothers for giving fluid during diarrheal illness

*Description *				
*Breast Milk*	*n=41*	*%*	*n=29*	*%*
No	20	48.78	6	20.69
Yes	21	51.22	23	79.31
Doesn't know	0	0.00	0	0.00
P-value	1	0.017		
*Cereal-based gruel or gruel made from roots or soup*				
No	19	46.34	8	27.59
Yes	22	53.66	21	72.41
Doesn't know	0	0.00	0	0.00
P-value	1	0.115		
*Other local home fluids (e.g., yoghurt drink)*				
*ORS Packet Solution*				
No	20	48.78	5	17.24
Yes	21	51.22	24	82.76
Doesn't know	0	0.00	0	0.00
P-value	1	0.007		
*Water with feeding during some part of the day*				
No	18	43.90	2	6.90
Yes	23	56.10	27	93.10
Doesn't know	0	0.00	0	0.00
P-value	1	0.001		
*Breast Milk*	n=41	%	n=29	%
No	31	75.61	6	20.69
Yes	10	24.39	23	79.31
Doesn't know	0	0.00	0	0.00
*P-value*	1	< 0.001		
*Other fluids but not feeding*				
No	29	70.73	23	79.31
Yes	12	29.27	6	20.69
Doesn't know	0	0.00	0	0.00
P-value	1	0.422		

As shown in Table-III the practice of giving more frequent breast feeding and use of ORS with diarrhea has been improved with statistically significant difference. Regarding the ability of the mothers to recognize the danger sign and when the sick child needs treatment outside the home, majority of mothers consider diarrhea, cough and malnutrition as major problem needing treatment outside the home. To assess mother ability to recognize the danger sign we asked; when should you take a sick child to a health facility right away? Mother’s response is shown in Table-IV.

**Table-IV:** When should you take a sick child to a health facility right away? (n=95)

*Problem*		*Baseline*	*Percentage*	*Post-intervention*	*Percentage*	*P-Value*
Child unable to drink or feed	No	86	90.53%	38	38.78%	< .000
	Yes	9	9.47%	60	61.22%	
Child becomes sicker	No	51	53.68%	20	20.41%	< .000
	Yes	44	46.32%	78	79.59%	
Child develops fever	No	52	54.74%	20	20.41%	< .000
	Yes	43	45.26%	78	79.59%	
Child has fast breathing	No	75	78.95%	41	41.84%	< .000
	Yes	20	21.05%	57	58.16%	
Child has difficult breathing	No	61	64.21%	32	32.65%	< .000
	Yes	34	35.79%	66	67.35%	
Child has blood in stool	No	39	41.05%	19	19.39%	<.005
	Yes	56	58.95%	79	80.61%	
Child is drinking poorly	No	87	91.58%	26	26.53%	< .000
	Yes	8	8.42%	72	73.47%	

The ability of mothers to recognize danger sign was improved during end-line survey with statistically significant difference (P-value <0.05). Further we asked about the time passed between recognizing the child’s illness and reaching to health facility? There was no statistically significant difference as 50% of replied in both surveys that they reach within 24 hours in the health facility.

Regarding the practices of mother to seek the treatment outside the home from an appropriate health care provider for their sick child, following question were asked from mothers during surveys. Mother’s response along with statistical analysis is given in Table-V.

**Table-V:** Practices of mother to seek the treatment from outside the home for their sick child

*Did you seek advice or treatment for Child illness outside the home?* *Baseline Post intervention*				
*Description*	*n=123*	*%*	*n=51*	*%*
No	44	35.77	2	3.92
Yes	79	64.23	49	96.08
P-value	1	< 0.001		^1^
*Whether caregiver sought care from formal, non-formal, or from both.*				
Formal	47	59.49	41	80.39
Non-Formal	32	40.51	10	19.61
P-value	1	0.013		^1^
*Is there someone in your community regularly consulted for childhood illness?*				
No	33	34.74	6	6.12
Yes	62	65.26	92	93.88
P-value	1	< 0.001		^1^
*With whom in your community do you regularly consult for childhood illness?*				
Spouse	0	0.00	0	0.00
Neighbour	0	0.00	1	1.09
Elder	0	0.00	1	1.09
Medical Doctor	37	59.68	88	95.65
Traditional healer	1	1.61	0	0.00
Religious Leader	0	0.00	0	0.00
Others (Medical Store / Pharmacy)	24	38.71	0	0.00
Not Reported	0	0.00	2	2.17
P-value	2	< 0.001		2

Regarding mothers ability to follow the health care providers advice; we enquire from the mother, did you find instructions of LHW easy to understand and follow? Majority >95% replied yes in both pre and post-intervention survey and the difference was not statistically significant. To know the mother’s exposure to the health facility and to know the mother’s practice to follow the doctors advice we asked few questions in relation to the last two weeks illness of their children. The questions along with the mothers response with statistical interpretation is given in Table-VI.

**Table-VI:** Did the health care provider ask you for follow up?

*Description*	*n=42*	*%*	*n=41*	*%*	
No	15	35.71	19	46.34	
Yes	27	64.29	22	53.66	
P-value	0.328				
*Did you bring the child back to the health facility for follow up?*					
No	15	55.56	5	22.73	
Yes	12	44.44	17	77.27	
P-value	1	0.021			
*Did the health care provider suggest for referral?*					
No	38	90.48	36	87.80	
Yes	4	9.52	5	12.20	
P-value	1	0.697			
*Were you able to comply?*					
No	3	75.00	1		20.00
Yes	1	25.00	4		80.00
P-value	1	0.190			

## DISCUSSION

Community based integrated approach is one of the most important strategies to improve child survival. Studies from developing countries have proved that educating mothers; the first care provider to the child, is essential to reduce child mortality.^10^

The extent to which the study results can be generalized depend upon the background characteristics of the population surveyed. In this study the respondents were mothers having children under five years of age. More than 90% of our mothers were housewives which is the situation in majority of our rural population. Status of polio was around 99% in both baseline and endline surveys, the community outreach services and media coverage for mass polio campaign may be the possible explanation for this achievement also shown in other studies as well.^11^

The immunization coverage for children in Pakistan varies from 56% to - 88% with considerable variation between provinces.

Contrary to other studies from Pakistan^12^, our study have shown higher rate of fully immunized children, though the rate of coverage was low [57%] for 3rd dose of pentavalent as shown in other national and international studies.^13^ Despite the fact that intervention area was covered by LHWs, EPI Programme is also followed by NPPI focal persons and twice a year 1week activity of Mother and Child week are celebrated with special emphasis on EPI. The target coverage was still less than 90% as targeted by MDGs. Mother’s perception and practices regarding childhood illness is very important for achievement of fourth and sixth Millennium Development Goals. Studies in developing countries have shown around 50% deaths from common illnesses like pneumonia and diarrhea occur at home, which means that recognition of illness by mothers, their knowledge and practices need to be improved to achieve reduction in child mortality. ^14^ We enquired from the mothers regarding current episode of illness in last 2-weeks to their child to assess hone care practices with particular emphasis to acute respiratory infection (ARI), fever, and diarrhea.

As the commonest illness was diarrheoa with 33% in the baseline survey and 56% in the post-intervention survey it was chosen as area for defining knowledge and practices of care takers and preparation of ORS as measurable factor for intervention. In our study, ORS (commercially available) was used by 51% in the baseline survey which improved to 82% in end-line. Regarding how to mix the ORS only 31% mothers knew the correct method which increased to 94% after intervention. According to national survey of Pakistan, the use of ORS in children under 5 years of age in different studies is 33%^15^ and 22%.^16^^,^

Oral rehydration along with appropriate feeding in children with diarrhea can markedly decrease the morbidity and mortality by 60%.^17^ Health education programs have been implemented throughout the world to encourage the use of oral rehydration solution. But In spite of widespread availability of ORS, many mothers continue to use other therapies or they use ORS incorrectly.^18^

Another important component of management of diarrheal diseases is appropriate feeding, especially for malnourished children.

In the management of diarrhea although rehydration therapy is a key to reduce mortality; appropriate feeding during and after convalescent from it is important to reduce malnutrition and its long term effects. However, across developing countries <25% of children with diarrhea consume increased food during illness. ^19^

Our study has also shown similar results that more mothers were aware of the importance of giving more fluid during diarrhea than more food, perhaps because of the media campaigns more on fluids than food for sick child.

A study from Lahore has shown that mothers are less resistance to offer more fluid than to give more food to sick child during diarrhoea.^20^ Continuous Behavioral Change Communication (BCC) strategy is required to bring a significant change in attitude and practices.

In a study by Khan MA et al.^21^, same diet as before diarrhoea was given in 59.9% of cases and in 40.6% of cases either feeding was stopped or reduced in quantity. Another study from Kosova revealed that almost half of the mothers 43.9% reduced or stopped, 48.6% gave usual amount and only 7.5% of them increased feeding or breast milk to children with diarrhea.^22^

Simple cost effective measures by up-scaling current interventions and strengthening existing systems can result in significant reduction of diarrhea mortality and worsening of malnutrition. ^23^

 Regarding the ability of the mothers to identify when the sick child requires treatment outside the home from an appropriate provider, majority of mothers consider diarrhea, cough and malnutrition as major problem. Mother’s the knowledge and awareness to recognize danger signs that indicate the child needs treatment outside the home is crucial in designing appropriate BCC strategies. ^24^

In this study mothers were inquired to spontaneously mention the warning signs that indicate a newborn and a child under the age of 5 years should be taken to a health facility. Overall, it appears that mothers were not sufficiently aware of those signs initially. It was measured as 35-58% in baseline survey but their awareness was improved to 70-80% in the post- intervention survey, similar findings have been documented by other researchers.^11^^,^^23^

Although the majority of the women (75%) reported that child with febrile illness should be referred to a health facility, but only 45% of the children suffering from fever were actually taken to health facility, indicating the contrast in the knowledge and practices of mothers.

Regarding the care seeking practices the behavior was improved to 96% in the post intervention from 64% in the baseline survey. By health education messages given during the intervention period mothers realized the importance to consult someone outside the home for their ill child. Timely recognition of key symptoms of illness by caregivers followed by seeking appropriate care is lifesaving. Care-seeking for children with symptoms of diarrhea and pneumonia has increased slightly in developing countries, from 54 percent around 2000 to 60 per cent around 2010.^11^^,^^22^ Even when mother thought that the child needs to be taken to a health facility, the lack of easy access, and the direct and indirect service cost may deter women’s ability to seek care.

Regarding the formal or non-formal health care provider, the frequent visits by doctors from university hospital encouraged mothers to seek care from hospital in OPD hours and privately in later hours. So after intervention 74% mothers responded to formal provider. Regarding the follow up care and follow-up visits mother’s compliance with health care provider’s advice improved from 9% baseline survey to 49% in the post-interventional survey.

All 5 key practices improved significantly after health education. Studies have shown that effects of such interventions do not last for longer period and require continued efforts if changes in attitude and behavior are to be sustained.^11^^,^^20^^,^^25^

The conclusion drawn from this intervention is that; repeated and focused health education messages through LHWs and media on the weaker area can improve the health status of our children in the community. Supervision by LHS, research officers and seniors during counseling sessions and random checking in community resulted in desired and timely delivery of messages.

## Recommendations:

- Maternal, newborn and child survival recline within households/families, communities and health institutions. There is need for interlocking efforts at all levels - households/families, community, health workers and health institutions. The synergy among these health resources, if well coordinated and managed, can advance rural health to the desired level.
- To make such BCC strategy workable and sustainable the health department needs to define proper monitoring and supervision system with targeted refresher courses and incentives depending on performance and health indicators of the area.
- Strengthening referral pathways, ensuring drugs availability in government hospitals may encourage people to use public instead of private facilities, reducing economic burden which results in vicious cycle of poverty and malnutrition.

## Authors contribution:

***Salma Shaikh:*** Contribution for conception and design, analysis and interpretation of data.

***Shazia Memon:*** Writing manuscript and Final revission of draft.

***Imran Ahmed:*** Collected and interpreted the data in out-patient department.

***Amna:*** Collected and interpreted the data during baseline survey.

***Rabia Manzoor:*** Study design and synopsis writing.

***Saleem Shaikh:*** Collected and interpreted the data during end-line survey.

## Notes

***Source of Funding: ***The study was funded by the PMRC, Islamabad. The sponsors had no part in the design, conduct, or analysis of the study.

## Notes

***Ethical approval:*** Technical advisory committee of PMRC had approved the project after analyzing all the ethical consideration.
